# System analysis with life cycle assessment for NiMH battery recycling

**DOI:** 10.1098/rsta.2023.0243

**Published:** 2024-11-04

**Authors:** Kivanc Korkmaz, Christian Junestedt, Nilay Elginoz, Mats Almemark, Michael Svärd, Åke C. Rasmuson, Kerstin M. Forsberg

**Affiliations:** ^1^ Department of Chemical Engineering, KTH Royal Institute of Technology, Stockholm SE-100 44, Sweden; ^2^ IVL Swedish Environmental Research Institute, Stockholm S-100 31, Sweden

**Keywords:** recycling, hydrometallurgy, rare earth elements, NiMH batteries, life cycle assessment

## Abstract

The nickel metal hydride (NiMH) battery technology has been designed for use in electric vehicles, solar-powered applications and power tools. These batteries contain the critical and strategic raw materials cobalt, nickel and several rare earth elements (REE). When designing a battery recycling process, there are several choices to be made regarding end-products and process chemicals. The aim of this study is to investigate and compare the environmental and economic sustainability of different recycling options for NiMH batteries by taking projected market developments into consideration and by applying life cycle assessment and life cycle costing methods. The comparative study is limited to recovery of the REEs. Two hydrometallurgical processes for recovery of the REEs from the anode material are compared with extraction of REEs from primary sources in China. The processes compared are a high-temperature sulfation roasting process and a process based on hydrochloric acid leaching followed by precipitation of REE oxalates. By comparing the different recycling approaches, the hydrochloric acid process performs best. However, the use of oxalic acid has a large impact on the overall sustainability footprint. For the sulfation roasting process, the energy, sodium hydroxide and sulphuric acid consumption contribute most to the total environmental footprint.

This article is part of the discussion meeting issue 'Sustainable metals: science and systems'.

## 1. Introduction

Rechargeable batteries are widely used in portable devices, electric vehicles, and energy storage systems. The nickel metal hydride (NiMH) battery technology has been designed especially for use in electric vehicles, solar-powered applications, and power tools. In 1989, NiMH batteries entered the market and started to replace the more environmentally hazardous Ni-Cd batteries in many applications. In the end of the 1990s, Toyota introduced NiMH battery packs in their Prius hybrid electric vehicles (HEV). Today, NiMH batteries in vehicles as well as many other applications are being replaced by lithium-ion battery (LIB) technology, which provides superior energy density and a longer cycle life. However, NiMH batteries remain competitive in certain applications, such as small and portable consumer and medical devices, due to their good performance in combination with high safety, low production costs and relatively long battery life. They are also able to fill niche roles such as outdoor applications in colder climates due to a larger operating temperature range. NiMH batteries contain the critical and strategic raw materials cobalt, nickel and rare earth elements (REEs), and various recycling processes have been developed to recover these materials to different extents and in different forms [1,2]. The European Union is actively working to improve the sustainability of batteries on the European market with the new batteries regulation put into force in 2023 and the batteries directive to be repealed by a new version in 2025 [3,4]. The REEs in particular have been declared critical raw material by the European Union, the United States and China, given their rapidly growing importance to many green technologies, leading to high demand and increasing supply risks [4–6]. In particular, contested supply of neodymium is emerging as an increasing threat to the green transition because of its dominant role in permanent magnets, a key component of electric motors and wind turbines [4].

There are still challenges with treating end-of-life NiMH batteries in a suitable way, both environmentally and economically [1,2,7]. Many efforts are focusing on the technical aspects of recycling NiMH batteries, from smaller-sized as well as larger-sized batteries, by pyrometallurgical and hydrometallurgical methods [1,2]. Industrially, NiMH batteries are commonly recycled by pyrometallurgical processes where the REEs end up in the slag when recovery is difficult. The pyrometallurgical recycling processes can handle large volumes but generally have a comparably high carbon footprint and low purity of products compared to hydrometallurgical processes [1,7–13]. Hydrometallurgical processes provide improved opportunity to recover all the valuable elements with higher purity. However, there are still several technical challenges remaining in current process schemes, including processes integration and scale-up [1]. Furthermore, environmental impacts should be considered in the design of the recycling processes [1]. To help with this, life cycle assessment (LCA) is a suitable tool to investigate and quantify the environmental impacts of different aspects of recycling processes. There are many examples of environmental sustainability assessments of batteries. In an early abridged LCA study [14], lead-acid (PbA), nickel-cadmium (NiCd), NiMH and sodium-sulfur (NaS) batteries were compared. According to this comparison, NiMH batteries had the least environmental burden, but as a disadvantage of NiMH batteries at the time (1998) Steele and Allen stated that there were no recycling technologies for NiMH batteries [14]. In line with the developments in battery technology, other comparative studies have since been conducted. The NiMH battery was found to have the highest environmental burden when compared to two types of LIBs (with nickel cobalt manganese (NCM) and iron phosphate (LFP) cathodes) in all environmental impact categories other than ozone depletion potential [15]. There were several reasons for this result; LIBs have a more efficient use phase, they store more energy during their life spans, they do not contain REEs and they contain less nickel. The study excluded the end-of-life phase of the batteries, and hence also the recycling step. When the end-of-life phase including recycling is included, it is stated that recycling end-of-life LIBs and NiMH batteries can help reduce their environmental burdens [7]. Wang *et al*. concluded that recycling of an NiMH battery instead of landfilling saves 83 kg CO_2_ [16]. However, the assumptions made in the LCA model for the recycling may have a substantial effect on the results [8]. Unterreiner *et al*. [9] investigated the cradle-to-cradle recycling of lead acid battery, LIB and vanadium redox flow battery technology. According to this study, the ecological impact of the batteries could be decreased by between 16 and 49% through recycling and reuse, depending on battery technology [9]. Production of REEs from primary sources was also investigated using LCA methodology. Vahidi and Zhao carried out an LCA of rare earth oxide (REO) production and concluded that 30% of the total burdens for the production of REOs originated from solvent extraction [10]. Collecting data for an LCA is time-consuming and sometimes assumptions have to be made. Another important aspect in the LCA of batteries in general is the rapid development of battery technology, which results in quick outdating of LCA results [8]. However, LCA can be a valuable tool in quantitative comparison of different recycling routes and in drawing general conclusions which are valuable in future process design.

Recovery processes of metals from waste batteries have also been investigated from a techno-economical point of view. Li *et al*. conducted a techno-environmental assessment for recovery of Co and Li from waste LIBs [16]. It is claimed that recovery of Co may be preferable to extracting Co from natural sources in terms of environmental impact. Gains and Dunn also suggest that recovery of LiMnO_2_ from waste lithium-ion batteries can consume less energy than producing cathode material from natural sources [12].

Despite the fact that several recycling routes for REEs have been developed to overcome REE shortage, there are only a few studies on the life cycle environmental impacts of these processes. Sprecher *et al*. investigated neodymium usage in high-performance magnets [13]. They compared the production of magnets from primary sources with two recycling processes and concluded that manual recycling of neodymium from computer hard disk drives has lower impacts when compared to primary production. In a comparative LCA study by Li *et al*. in 2019, recovery of REEs and precious metals from electronic waste using a novel electrochemical recovery process was compared to two technologies based on pyrometallurgical and hydrometallurgical processes, and it is claimed that the environmental performance of the electrochemical process is better than for the other two methods [11]. In a recent study [17], a hydrometallurgical recycling process for a mix of LIB and NiMH batteries to recover metals including Ni, Co and REEs was investigated [17]. The results indicated that the recycling process has a lower environmental impact in four categories (climate change, acidification, freshwater eutrophication and human toxicity) when compared to primary production of battery metals. Despite the lack of sufficient studies on the topic, recycling of REEs is expected to lead to environmental benefits; however, this assumption must be investigated through a scientific approach [5].

In this work, LCA has been applied in the early design stage of two recycling routes to draw conclusions that are valuable in the development of sustainable recycling processes, both for NiMH battery recycling specifically but also in more general terms considering process operation conditions and chemical usage.

## 2. Data and methodology

For LCA modelling, the GaBi software with its databases (now called ‘LCA for Experts’ owned by the company Sphera) was used with modifications for some of the processes. LCA is a quantitative method to define environmental impacts and resource consumption of a system from cradle to grave. Although high data demand for material and energy flows makes LCA harder to use in the design stage, it is still appropriate for quantitative analysis. Therefore, LCA methodology in line with ISO Standards 14044:2006 has been used in this study. An LCA comprises four main stages: goal and scope definition, life cycle inventory, life cycle impact assessment and interpretation.

### (a) Aim and scope

**Figure 1.**
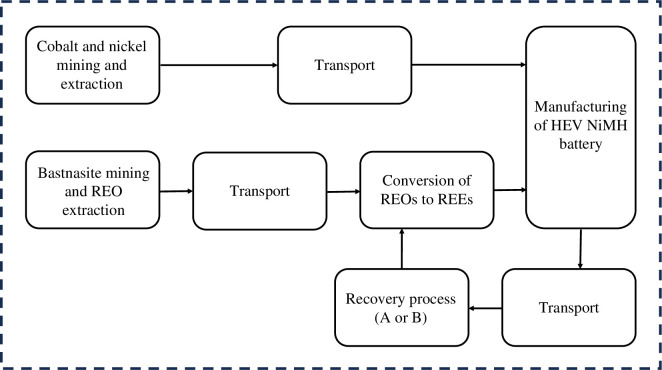
System boundary.

The aim of this study is to assess and compare the environmental and economic sustainability of different NiMH battery recycling options. Two different hydrometallurgical processes for REE recovery from HEV NiMH anode material are considered, and the manufacturing of HEV batteries using either recycled or primary REEs are compared. The Functional Unit is to supply the manufacture of 1 HEV batteries with the necessary quantities of rare earth metals, nickel and cobalt. The first recycling process considered (A) is based on a high-temperature sulphate roasting followed by water leaching of REE sulphates, and the second recycling process (B) is based on hydrochloric acid leaching followed by precipitation of REE oxalates, see §2.3 for detailed process descriptions. The high-temperature sulfation roasting process and the oxalate precipitation process options were chosen because they both result in REE oxides as final product in fewer steps as compared to e.g. selective precipitation of rare earth double sulfate salts, which is another viable method [2,18]. During the production of HEV batteries, Ni and Co are used in addition to REEs and other materials like manganese, aluminium, etc. However, the usage of the latter elements is identical for each considered case; therefore, the effect of these materials is excluded from the study. Since the processing of oxides to metals (acid dissolution, molten-salt electrolysis, etc.) can be considered to be equivalent whether the REOs come from ore or via a recycling route, these processes are also excluded in this study. In addition, the waste streams from the production of the battery are the same in each case and are therefore also excluded. The boundary of the investigated system is given in figure 1. The grinding of material is included in the LCA while upstream battery disassembly and separation are excluded.

The module ‘Conversion of REO to REE’ is just a recalculation of the mass flows from the oxides to the metals. The reduction process is not included in the system. The new REE recovery routes investigated in this study are supposed to apply within Europe. Therefore, European data were used for the recovery processes. The mining of REO takes place in China, so Chinese and/or global data was used for the extraction processes. For transport, solely global data was used. The data sets collected from the databases were modified and economic allocations were used to be compatible with the other datasets.

#### (i) Impact assessment method

The CML (Center for Environmental Science, University of Leiden) 2001 methodology January 2016 version was used for classification and characterization. The chosen impact categories for LCIA are global warming potential (GWP), acidification potential (AP), eutrophication potential (EP), photochemical ozone creation potential (POCP) and abiotic depletion potential for Fossil Energy Resources (ADP fossil). The calculation for ADP could not be conducted due to the absence of characterization factors for all of the REE.

#### (ii) Allocations

Different (sub-) processes are allocated to use of resources as well as emissions in specific cases. The LCI data sets, which contain different REO, were gathered from various databases and modified/adjusted to be used in this study. In addition to that, economic allocations were also recalculated and used for the LCA. Information regarding inventory data can be found in the electronic supplementary information.

### (b) Cost assessment

The life cycle costing (LCC) approach has been used for the cost assessment. Capital cost, maintenance, labour and operation cost calculations were used to assess the leaching system. The cost was categorized as lifetime annuities which correspond to regular and annual payments over a limited period of time. The net present value remains the same, which is the sum of all costs and receipts at a given point of time, adjusted with a specific discount rate.

Initially, the net present value is calculated as follows:


(2.1) $$LCC_{tot}=\sum_{t=0}^{t=n}\frac{C_{t}}{{\left(1+r\right)}^{t}},$$

where *n* is the considered economic life, *r* is the interest rate and *C_t_* are the calculated total costs at time *t*. These costs are then multiplied by an annuity factor:


(2.2) $$\frac{LCC}{year}=LCC_{tot}\times \frac{r}{1-{\left(1+r\right)}^{-f_{n}}},$$

where *f_n_* is the economic life of the core process facility. An annuity can be connected to functional units. Cost calculations for the peripheral process were not conducted. However, they are included in the operational costs.

### (c) Investigated systems

The system investigated in this study is the manufacturing and recycling of HEV NiMH batteries. In the manufacturing of HEV NiMH batteries, REEs (cerium, lanthanum, neodymium, praseodymium and yttrium), manganese, aluminium, iron, potassium, zinc, nickel and cobalt are used. The HEV battery consists of 168 cells. Each cell is comprised of 25 plates, of which 12 are cathodes and 13 anodes. The anodes contain most of the REEs. The chemical composition of anode and cathode materials in mass percentage is taken from literature [18]. The total amount of REEs, Ni and Co and some other metals in the anode and the cathode of one HEV battery are listed in electronic supplementary material, table S1.

In the base case, it is assumed that all REOs are extracted from bastnasite ore from the Bayan Obo mine in China, transported to Europe, refined into REEs, and used in NiMH HEV battery production. The process is conducted in several steps; leaching bastnasite with hydrochloric acid, concentrating the output by froth flotation, roasting at 500°C with sulfuric acid, separating the REEs by solvent extraction, transforming to chlorides using hydrochloric acid, electrolysis and cathodic reduction. The logistics of the base case from the mine to the battery manufacturing facility was broken down into three steps; 700 km from the mine to Tianjin by truck, 21 000 km from Tianjin to Hamburg by bulk carrier and 300 km from Hamburg to the manufacturing site by truck.

The base case is compared with three cases in which the REEs are recycled by different methods from HEV NiMH batteries. Recycling of REEs was modelled as a closed recycling loop in the LCA model.

The manual deep discharge, dismantling and treatment to recover the anode material from the waste NiMH battery is reported by Korkmaz *et al.* [18]. The upstream treatment to recover the anode material is identical for the investigated hydrometallurgical recycling processes. In recovery process (A), sulphate roasting is used followed by water leaching of REE sulphates. The second recycling process (B) is based on hydrochloric acid leaching followed by precipitation of REE oxalates with either 100% of the stoichiometric demand of oxalic acid (Ba) or 300% of the stoichiometric demand of oxalic acid, to increase the yield of REE (Bb), see table 1.

**Table 1.** Summary of the investigated systems.

base case	primary REO exported from Bayan Obo mine China
Recovery A	drying and roasting with H_2_SO_4_ and leaching with water
Recovery Ba	leaching with HCl and precipitation by oxalic acid (100%)^a^
Recovery Bb	leaching with HCl and precipitation by oxalic acid (300%)

^a^ Stoichiometric excess of oxalic acid to total REE.

In the LCA model, REOs are considered instead of REEs. Conversion of REOs to REEs are identical in production from primary sources and in recycling. Nickel and cobalt consumptions were also considered in the manufacturing of the batteries. These metals can also be recovered from the batteries; however, their recovery was not included in the study since the scope of the study is limited to compare the respective recovery of REEs.

**Figure 2.**
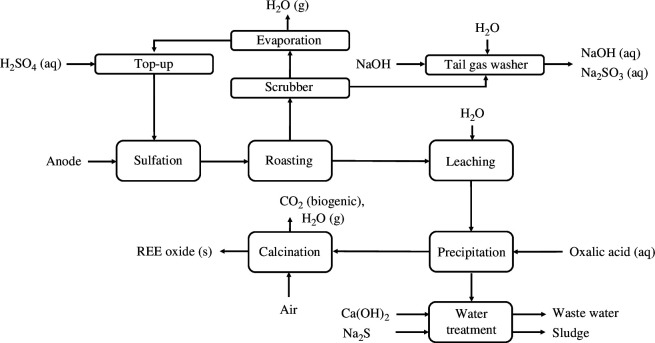
Block flow diagram of the sulfation, roasting and REE water leaching process (A).

The sulphate roasting process (A) to recover REE from anode material is presented in figure 2. The process starts with an exothermic sulfation process conducted with 8M sulfuric acid initially at room temperature to transform the metals into their sulphate complexes according to the following reactions:


(2.3) $$aM+2bH_{2}SO_{4}\left(aq\right)\to M_{a}{\left(SO_{4}\right)}_{b}\left(aq\right)+bSO_{2}\left(g\right)+2bH_{2}O\left(g\right),$$

where *M* denotes metal (REE, Al, Co, Ni, Fe, Mn, Zn, K), *a* = 2 and *b* = 3 for REE, Al (trivalent ions), and *a* = *b* = 1 for Co, Ni, Fe, Mn, Zn (divalent ions). After the sulfation process, the mixture is roasted in a furnace at 850°C for 2 h. This selective roasting allows all elements to be transformed into metal oxides, except the REEs which remain as sulphates. During the process, sulphur dioxide and trioxide evolve as well as water vapour. Flue gases are dealt with by neutralizing SO_2_ with NaOH into Na_2_SO_3_. The SO_3_ from the roasting process is recovered by reaction with H_2_O and is recycled as H_2_SO_4_. The solid mixture collected from the roasting is leached with water at ambient temperature for 1 h. Because of the high solubility of REE sulphates in water, selective leaching of the REEs to separate them from the insoluble metal oxides is possible. The leach residue collected from the water leaching contains a negligible amount of REOs together with high amounts of nickel and cobalt in oxide form. The leach residue could be considered as a byproduct. A more detailed technical description of the process can be found in literature [19].

**Figure 3.**
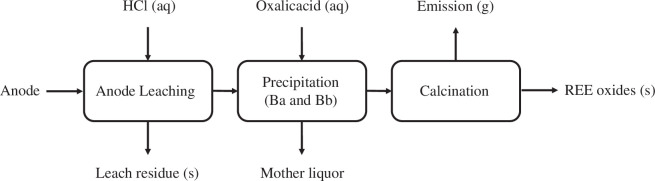
Block flow diagram of the HCl leaching and REE oxalate precipitation process (B).

The second REE recovery process (B) is based on leaching the mechanically separated and pulverized anode material with 1 M HCl at 25°C for 2 h, followed by precipitating the REEs with oxalic acid, see figure 3. In the first scenario (Ba), the amount of oxalic acid was chosen to correspond to the stoichiometric amount necessary to precipitate all REE content. This resulted in recovery of about 75% by mass of the REEs as REE_2_(C_2_O_4_)_3_·nH_2_O with a purity of 99.9%. In a second scenario (Bb), 300% excess of oxalate compared to the amount in the first scenario was used. In this scenario, 95% by mass of the REEs are recovered as REE_2_(C_2_O_4_)_3_·nH_2_O with a purity of 90.1% due to co-precipitation of mainly nickel oxalate [18]. The calcination of the oxalate is conducted in a furnace at 450°C with capabilities of discharging the evolving gases. The solid product from the calcination is a mixture of REOs. The details of the composition of the starting material, leaching process parameters and precipitation process are adopted from a published study [18].

Laboratory-scale data for processes (A) [19] and (B) [18] were used as the basis for preliminary engineering designs of full-scale processes. The mass balances are based on the results of the laboratory experiments, supplemented with calculations and some assumptions. The mass flows have been recalculated to the treatment of 1 anode. The laboratory experiments were carried out with 100 g anode samples. The necessary upscaling calculations were carried out, such as heat and electricity consumption, industrial-grade containers to handle the proposed processes, supporting units (heat exchangers, scrubbers, dosing units) and all chemicals used in the main or the side operations to run the process according to health, safety, and environmental concerns.

**Figure 4.**
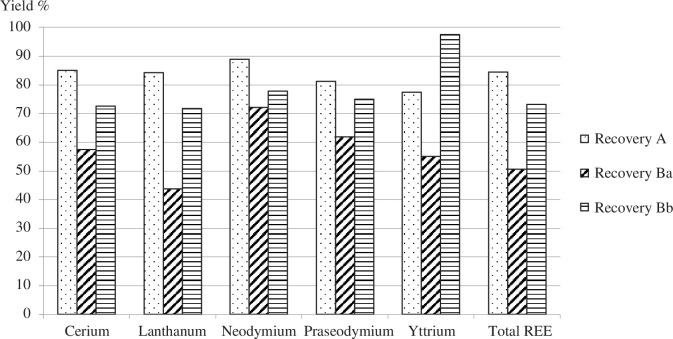
Yields of recovered REEs from the anodes of an NiMH battery.

The yields of REE recovery in each case are given in figure 4, including an assumed 10% loss of metals in the upscaled process. Overall, recovery process (A) based on sulfation roasting provides the best yield in terms of REEs.

## 3. Results and discussion

### (a) Life cycle impact assessment

**Figure 5.**
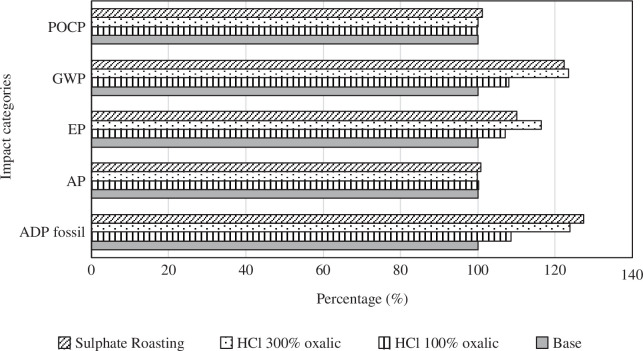
Table with cumulative results for the base case and all recovery processes (A), (Ba) and (Bb). All values are normalized against the base case value within each impact category.

The main results for each case; the base case, and recovery processes (A), (Ba) and (Bb) are given in table 2, and a comparison of their impact assessment on the manufacturing of 1 HEV battery is given in figure 5. For the categories AP and POCP, the results for each case are almost the same, so neither recovery processes nor the base case comes forward in terms of these two categories. For GWP, EP, and fossil ADP categories, the base case shows a better performance, but this result does not prevent the need for new recycling routes. The main objective of the present study is to compare the recycling routes for REEs, not the overall recovery processes. Therefore, comparison of the new recycling routes internally will give results that are more meaningful in the scope of this study. For the GWP, EP and ADP fossil categories, recovery process Ba shows the best performance. In the GWP category, recovery processes A and Bb show very similar results, 124, and 122%, respectively, with recovery process Bb having slightly higher impacts, normalized against the base case (at 100%). In the EP category, recovery process Bb has higher impacts than recovery process A, 116 and 110%, respectively, and for ADP fossil category, recovery process A has higher impacts than Bb, 127 and 124%, respectively. According to overall results, recovery process Ba is the best route in terms of environmental impacts. Increasing the stochiometric oxalic acid equivalent from 100% to 300% in the HCl leaching route (B) increases the recovery yield, but this increase in recovery yield does not compensate for the increased environmental burdens. However, the difference between the three routes is less than 20 percentage points.

**Table 2.** Life cycle impact assessment results of manufacturing 1 NiMH HEV battery.

impact category	unit	base	recovery (A) sulphate roasting	recovery (Ba) HCl 100% oxalic	recovery (Bb) HCl 300% oxalic
ADP fossil	MJ	2.15E + 03	2.75E + 03	2.34E + 03	2.67E + 03
AP	kg SO2 eq.	3.76E + 01	3.79E +0 1	3.77E + 01	3.76E + 01
EP	kg phosphate eq.	1.49E + 00	1.64E + 00	1.60E + 00	1.74E + 00
GWP	kg CO2 eq.	1.98E + 02	2.43E + 02	2.14E + 02	2.45E + 02
POCP	kg ethene eq.	1.55E + 00	1.57E + 00	1.55E + 00	1.55E + 00

When we focus on the share of recovery processes in total impacts from the recovery system, the sulfation process causes 1190 MJ ADP, HCl leaching with 100 and 300% oxalic acid cause 587 and 1060 MJ, respectively. EPs of sulphating, HCl leaching with 100 and 300% oxalic acid are 0.314, 0.209 and 0.399 kg phosphate eq. GWP of the routes A, Ba and Bb are 91.5, 47.3 and 89.3 kg CO_2_ eq.

### (b) Contribution analysis and interpretation

**Figure 6.**
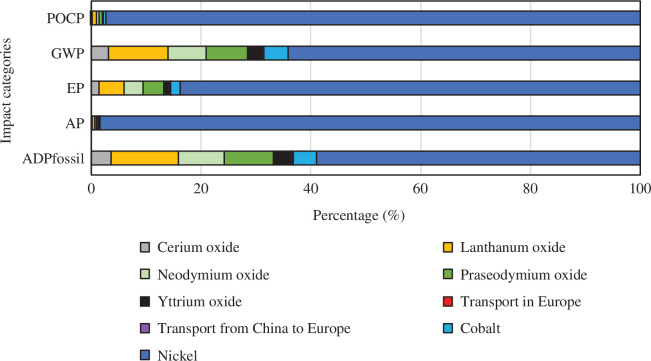
Contribution graph for the base case.

**Figure 7.**
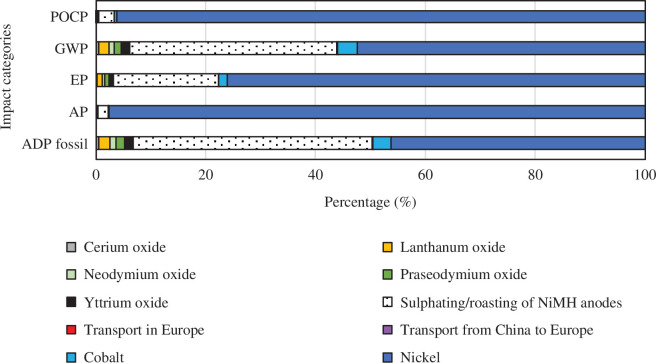
Contribution graph for recovery process A, sulphate roasting.

**Figure 8.**
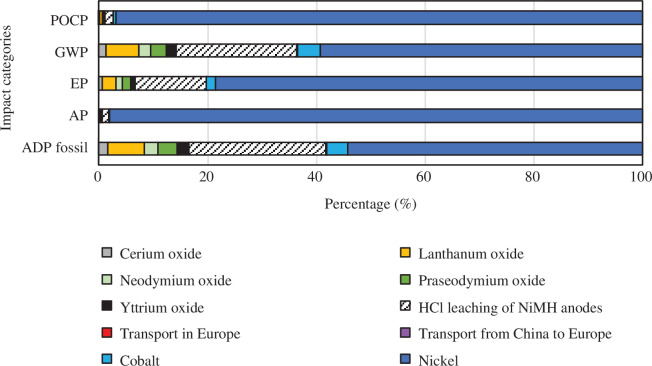
Contribution graph for recovery process Ba, HCl leaching (100% oxalic acid).

**Figure 9.**
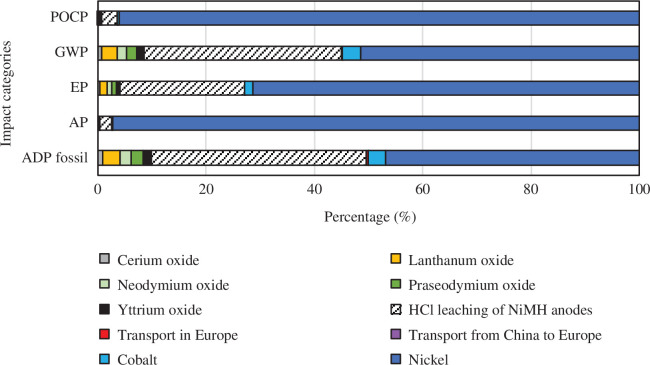
Contribution graph for recovery process Bb, HCl leaching (300% oxalic acid).

Contributions of sub-processes to total impacts for each case are given in figures 6–9. The values for the REOs refer to the impacts of producing from ores the quantities of oxides necessary to replace the recovery losses. In all cases and all categories, nickel has the highest share, and transport from China to Europe and transport within Europe have insignificant shares. In the base case, REEs share the rest of the impacts. In recovery process A, the sulfation process contributes very little, 2% and 3% to POCP and AP, respectively. However, it has a significant contribution to the GWP (38%), EP (19%) and ADP fossil (43%) impact categories. A similar pattern is seen for recovery process B (a and b). The HCl leaching 100% oxalic acid case (Ba) has a share of 22% GWP, 13% EP, and 25% ADP fossil, and the HCl leaching 300% oxalic acid case (Bb) has a share of 36% GWP, 23% EP and 40% ADP fossil.

**Figure 10.**
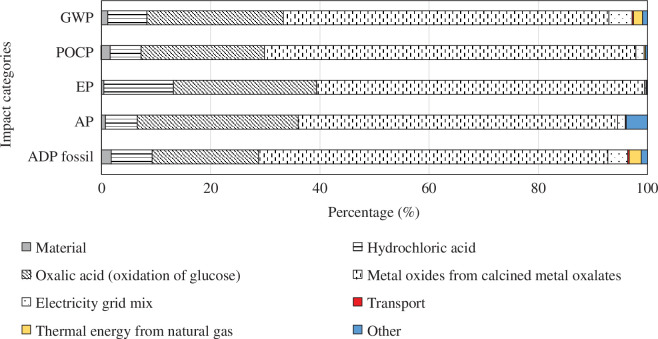
Contribution graph for HCl leaching of NiMH anodes (100% oxalic acid), process Ba, comparing major contributing factors.

In the HCl leaching 100% oxalic acid case (Ba), the share of metal oxide separation varies between 60 and 68% for all impact categories and oxalic acid contributes between 19 and 29%, see figure 10. In the recovery process Bb (HCl leaching 300% oxalic acid) a similar pattern as for Ba (100% oxalic acid) is observed with an expected increase in the share of oxalic acid. Metal oxide separation contributes between 48 and 59%, and oxalic acid contributes between 32 and 44% to all impact categories.

**Figure 11.**
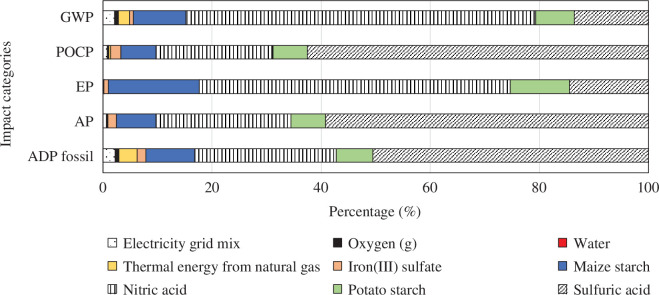
Contribution graph for oxalic acid production.

Oxalic acid has a big impact on all categories. During the production of oxalic acid, sulfuric acid is used to hydrolyse starch, nitric acid is used to oxidize glucose, and both cause high environmental burdens. The contribution graph for this sub-process is presented in figure 11. If waste sources are used to obtain glucose instead of starch, and if nitric acid is replaced by a more environmentally friendly oxidant, the environmental performance of the recovery process could be improved.

**Figure 12.**
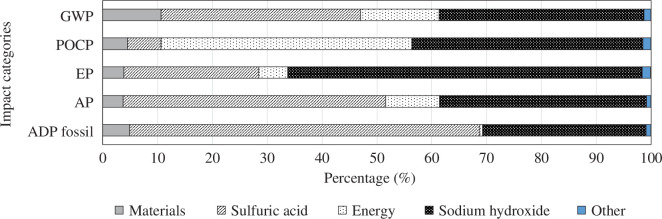
Contribution graph for sulfation roasting of NiMH anodes, process A, comparing major contributing factors (excluding the oxalate precipitation step).

In the sulfation roasting process (A), the metal oxide separation by oxalate precipitation followed by calcination contributes 67% to the total GWP. Excluding the oxalate precipitation and calcination and focusing only on the sulfation roasting process, the largest shares in the total GWP are sodium hydroxide (37%), sulphuric acid (36%), energy (14%) and materials (11%), see figure 12. For the other categories (POCP, EP, AP and ADP fossil) sodium hydroxide, sulphuric acid and energy use also have the biggest impacts.

In all cases (the base case and the recovery processes), separation of oxides from the mixture causes significant burdens. In the hydrochloric acid-based processes (B) the oxalic acid usage is the most important contributor, while in the sulphate roasting process (A) the sodium hydroxide, sulfuric acid and energy demand are the most important contributors. The separation of the REEs, conversion from oxides to metals and oxide concentrate preparation are important contributors in the REE metal production.

### (c) Cost assessment

The LCA results indicate that the cost of retrieving the REEs from any of the investigated processes is higher than the cost of the same elements available in the market. The primary reasons for high costs are the usage of high amounts of acids and high temperature in addition to costs for materials used in the initial equipment purchase with relatively low service time due to the processing conditions. In table 3, costs of the items are presented with their per cent share of the total cost during the 20 years of economic life for the two investigated processes A and Ba. The major cost for the roasting method is the labour cost due to 24 h’ operation per day. In the case of the oxalic acid process (Ba), replacement costs are the highest. The general costs for power and consumables are relatively low due to assumptions made in the way plants are operated and the length of service life.

**Table 3.** Percentage distribution of the costs for the two investigated routes.

cost	investment	replacement	power and consumable	labour
sulfation roasting (A)	12%	18%	15%	54%
HCl, oxalic acid (Ba)	24%	36%	13%	27%

The comparison of recycling costs for one anode to the market value of the recovered quantities of REOs is presented in table 4. The prices for market values have been recalculated to EUR from Chinese renminbi. The cost of separation of the REO mixture into individual REOs was not included in the reported recovery costs; still the value of the recovered REE does not cover the operating costs of the recovery process. However, the value of nickel and cobalt and the value revenue from them are not included in this analysis. Furthermore, the market values of the metals are fluctuating over time. The cost assessment mainly contributes to the comparison of the chosen REE recycling processes to each other and to REE recovery from the ore in general terms.

**Table 4.** The cost of recovered REOs from one anode compared to market values.

	market value^a^ EUR/kg	quantities recovered from 1 anode after separation, kg		market value of the recovered oxides, EUR		recycling costs, EUR/anode	
Process	—	Ba	A	Ba	A	Ba	A
Nd_2_O_3_	34	0.15	0.18	5	6	—	—
La_2_O_3_	1	0.66	1.28	1	2	—	—
Ce_2_O_3_	1	0.27	0.40	0.4	0.6	—	—
Pr_2_O_3_	42	0.12	0.16	5	7	—	—
Y_2_O_3_	2	0.03	0.04	0.06	0.09	—	—
Total:	—	—	—	11.5	15.7	44.0	92.0

^a^ Ref. [20].

The most energy-efficient conditions for the sulphation process (A) were assumed to be running the roasting process (at 850°C) and calcination process (at 450°C) at constant temperatures for 24 h per day. This method decreases the cost for energy at the expense of higher labour cost. The interest rate in this study is set to 4% and was hypothetically lowered to 1% to be able to see the difference in cost per anode, which was a few euros. The reason for this is the high cost for replacements and labour. There is still room for improvements regarding the way the plants might be operated if designed in reality, and the accuracy of the data used could have been of a better quality if a pilot scale plant had been used as an intermediate step between the laboratory trials and the theoretically designed full-scale plants.

### (d) Concluding discussion

Technology shifts complicate the design of recycling processes. New battery chemistries are constantly being developed. Successful recycling can reduce the environmental footprint of the batteries and reduce waste. However, developing a sustainable recycling process is far from trivial; there are multiple choices of process conditions and chemicals. In this work, LCA was used to systematically compare different aspects of two chosen recycling approaches, focusing especially on the recovery of the REEs from NiMH anode material. There are several studies pointing out the imbalance between the upstream supply and the downstream disposal of key REEs from NiMH batteries as well as other REE-containing waste streams, and as a consequence the EU is heavily reliant on import [21]. Recycling of REEs can reduce the supply risk and has been claimed to decrease the environmental burdens from producing REEs from ores [5]. However, the results of this study show that the environmental burdens of REE recovery by the investigated recycling processes can be considerable. With due caveats for assumptions, uncertainties and non-optimized processes, the burdens of some impact categories are shown to exceed those of REE mining, even if the total environmental burdens of the base case and the hydrochloric acid-based recovery process (Ba) are quite close. However, even if supply risk is not considered, based on these results it is not possible to claim that REE recovery is unprofitable for the environment. There are mainly two factors to be considered further: the economies of scale and the learning curve. Overall, environmental burdens are expected to decrease at full scale and with time. Furthermore, the results show that, both with and without REE recovery, by far the main contributor to the total environmental impact is nickel consumption – hence, further research focusing on LCA of combined REE and Ni recovery from waste NiMH batteries is recommended.

Due to variations in system boundaries and functional units, direct comparisons with previous studies are complicated but still useful. Majeau-Bettez *et al*. compared the environmental impacts of NiMH batteries with NCM and LFP batteries [15]. The functional unit of the study was storage of 50 MJ energy but to compare their results with other studies they also calculated their results for a 1 kg battery. The results show that the production of 1 kg NiMH battery causes 20 kg CO_2_ eq GWP. To make a comparison with Majeau-Bettez *et al*., the results of this study were divided by the total mass of the covered constituents. In the present study, the GWP for production of a 1 kg battery in the base case and with recovery processes A, Ba and Bb are 13.78, 16.86, 14.88 and 17.02 kg CO_2_ eq., respectively. Thus, the results of this study are similar to the study by Majeau-Bettez *et al*.; however, it is important to acknowledge that the previous study includes all the components, which constitute a single HEV battery. The present study only covers REEs, nickel, cobalt and their transport, as the focus is to compare different recycling routes for supplying REEs with manufacturing using primary resources.

The two processes chosen for this study were based on using either sulphuric acid (process A) or hydrochloric acid (process B). Sulphuric acid is less corrosive and has a lower market price than hydrochloric acid, which was partly reflected in the cost assessment. Today, more than 80% of the global sulphur supply originates as a byproduct from the oil and gas industry [22]. In the future, decarbonization will lead to a reduced production of fossil fuels and subsequently a decreased supply of sulphur. Sulphur can also be sourced from mining [23]. Hydrochloric acid is mainly produced as a byproduct of the production of other chemicals, and the majority of HCl originates from combustion of organic compounds, both involving non-renewable and renewable sources and relying on the chlor-alkali process for chlorine production from brine [24]. The LCA is based on current production methods and the present study identifies the hydrochloric acid-based process (Bb) as the most promising, not primarily because of the environmental footprint of mineral acids but rather the combined sodium hydroxide and energy demand for the sulfuric acid-based process (A). Regarding process chemical use an interesting finding from the study is the environmental footprint of oxalic acid and its impact on the LCAs. Oxalic acid is a common chemical used to precipitate REEs, which can then be calcined into a REO product, as in the present study. Furthermore, oxalic acid has been put forward as a green chemical in recycling of lithium-ion batteries [25]. The high environmental impact of oxalic acid stems from the chemical use in the current production process. If waste sources are used to obtain glucose instead of starch, and if a more nature-friendly oxidant could be used, the environmental performance of the recovery process could be improved. The importance of organic acid production methods on the sustainability of battery recycling processes was highlighted by Li *et al.*, who also stressed the importance of acid recycling in order to reduce the full fuel cycle energy intensity of the process [11].

## 4. Conclusions

The overall results show that the extraction of REEs from primary sources in China performs better in GWPglobal warming, EP and ADP and performs similarly in acidification and POCPs compared to REE recovery from the waste NiMH active anode material in Europe by the proposed recycling processes. Transport from China to Europe and transport within Europe have insignificant shares.

In the process based on hydrochloric acid leaching followed by precipitation of an REE oxalate concentrate, the oxalic acid usage is the most important contributor to reduced sustainability. In the sulfation roasting process, the sodium hydroxide, sulfuric acid and energy use are the most important contributors. The LCA identifies the hydrochloric acid-based process including precipitation of an REE oxalate concentrate as the most promising approach. The study identifies a high environmental footprint of oxalic acid, which has a significant impact on the LCAs. If waste sources are used to obtain glucose instead of starch and if a more nature-friendly oxidant could be used, the environmental performance of the recovery process could be improved.

The main contributor to environmental impacts is nickel consumption, so future research is recommended to focus on LCA of combined REE and nickel recovery from waste NiMH batteries. If the process residues, the leach residue and the supernatant from the oxalate precipitation could be utilized to recover nickel and cobalt in a way that is environmentally more efficient than the extraction of these metals from ores, this could make the recovery process as a whole better than the sourcing of the metals from primary sources.

## Data Availability

Supplementary material is available online [26].
